# The *Drosophila* homolog of *APP* promotes Dscam expression to drive axon terminal growth, revealing interaction between Down syndrome genes

**DOI:** 10.1242/dmm.049725

**Published:** 2023-09-15

**Authors:** Sarah Pizzano, Gabriella R. Sterne, Macy W. Veling, L. Amanda Xu, Ty Hergenreder, Bing Ye

**Affiliations:** ^1^ Life Sciences Institute, University of Michigan, Ann Arbor, MI 48109, USA; ^2^ Neuroscience Graduate Program, University of Michigan, Ann Arbor, MI 48109, USA; ^3^ Department of Biomedical Genetics, University of Rochester Medical Center, Rochester, NY 14642, USA; ^4^ Cellular and Molecular Biology Program, University of Michigan, Ann Arbor, MI 48109, USA; ^5^ Department of Cell and Developmental Biology, University of Michigan, Ann Arbor, MI 48109, USA

**Keywords:** Amyloid precursor protein, Down syndrome cell adhesion molecule, APP, Dscam, Down syndrome, *Drosophila*, Axon

## Abstract

Down syndrome (DS) is caused by triplication of human chromosome 21 (HSA21). Although several HSA21 genes have been found to be responsible for aspects of DS, whether and how HSA21 genes interact with each other is poorly understood. DS patients and animal models present with a number of neurological changes, including aberrant connectivity and neuronal morphology. Previous studies have indicated that amyloid precursor protein (*APP*) and Down syndrome cell adhesion molecule (*DSCAM*) regulate neuronal morphology and contribute to neuronal aberrations in DS. Here, we report the functional interaction between the *Drosophila* homologs of these two genes, *Amyloid precursor protein-like* (*Appl*) and *Dscam* (*Dscam1*). We show that *Appl* requires *Dscam* to promote axon terminal growth in sensory neurons. Moreover, *Appl* increases Dscam protein expression post-transcriptionally. We further demonstrate that regulation of Dscam by *Appl* does not require the Appl intracellular domain or second extracellular domain. This study presents an example of functional interactions between HSA21 genes, providing insights into the pathogenesis of neuronal aberrations in DS.

## Teaser

**Summary:** Down syndrome is caused by an extra copy of human chromosome 21 (HSA21). This study reveals that the interaction between two HSA21 genes is important for axon development.

## INTRODUCTION

Down syndrome (DS) occurs at a prevalence of 1 in approximately every 700 live births in the United States (Canfield et al., 2006). DS is caused by full or partial trisomy of human chromosome 21 (HSA21), resulting in increased RNA and protein expression of many of the triplicated genes in DS patients (Cheon et al., 2003; Olmos-Serrano et al., 2016). This large-scale genetic dysregulation causes a range of symptoms, including intellectual disability, congenital heart defects and Alzheimer's disease (AD) (Janicki and Dalton, 2000).

Although individual genes that are responsible for some aspects of DS have been identified (Korbel et al., 2009), whether and how HSA21 genes interact with each other is poorly understood. We previously reported that the *Drosophila* homolog of the HSA21 gene Down syndrome cell adhesion molecule (*DSCAM*), termed *Dscam1* (referred to as *Dscam* hereon), promotes axon terminal growth in a dose-dependent manner (Kim et al., 2013; Wang et al., 2013). Although *Drosophila* Dscam proteins have a remarkable molecular diversity derived from extensive alternative splicing (Schmucker et al., 2000; Zipursky and Grueber, 2013; Zipursky and Sanes, 2010), the protein-level-dependent regulation of presynaptic arbor size does not require the molecular diversity of Dscam in *Drosophila* (Kim et al., 2013). Similarly, mammalian DSCAM does not have extensive molecular diversity, and studies of mouse optic pathway development suggest that the rodent DSCAM is also required for axonal growth in retinal ganglion cells (Bruce et al., 2017). Furthermore, gain of *DSCAM* leads to precocious growth of retinal ganglion cell axons. These studies suggest a conserved role for DSCAM/Dscam in presynaptic arbor growth from insects to mammals. Because the protein levels of mammalian DSCAM are elevated in the brains of DS animal models and patients (Baumann, 2007; Saito et al., 2000), the axon terminal overgrowth caused by increased Dscam levels led to the hypothesis that elevated DSCAM levels drive changes in axon terminal growth in DS animal models and patients. This seems to be the case, at least in the cortical chandelier cells in the Ts65Dn DS mouse model (Liu et al., 2023).

Several lines of evidence suggest that other molecules interact with Dscam to regulate presynaptic arbor sizes. First, overexpression of a Dscam mutant that lacks most of the cytoplasmic domain (DscamΔCyto) appears to act as a dominant negative, decreasing the phosphorylation of Abelson tyrosine kinase (Abl) below wild-type levels (Sterne et al., 2015). The dominant effect of DscamΔCyto raises the possibility that other molecules are involved in this pathway. Second, whereas Abl mediates Dscam regulation of axon terminal growth (Sterne et al., 2015), Abl is known to promote axon growth in several other systems and to interact with cell adhesion molecules other than Dscam, suggesting that several separate or intertwined pathways may converge on Abl to control presynaptic arbor size (Leyssen et al., 2005; Soldano et al., 2013). Two scenarios could be imagined. Multiple upstream molecules could act separately to control Abl activation, interacting only through their convergent influence on Abl. Alternatively, Dscam could cooperate with another membrane protein as co-receptors to modulate Abl activity together. *Amyloid precursor protein-like* (*Appl*), the *Drosophila* homolog of human amyloid precursor protein (*APP*) (Rosen et al., 1989), has been shown to require Abl to promote axon and presynaptic arbor growth in two different *Drosophila* systems (Leyssen et al., 2005; Soldano et al., 2013). *APP* is triplicated and has increased expression in DS patient neurons (Matsui et al., 2007; Oyama et al., 1994), and this increase in expression alters synapse formation and axon patterning during development in primary neuronal cultures, flies and mouse DS models (Hoe et al., 2012). We thus wondered whether Dscam might interact with Appl to control presynaptic arbor growth.

In this study, we show that *Appl* and *Dscam* interact to regulate the growth of axon terminals during development. Co-overexpression of *Appl* and *Dscam* increased axon terminal length more than expressing either transgene independently. We found that *Appl* promotes axon terminal growth by upregulating the expression of Dscam. Appl increases the level of Dscam protein, but not that of the transcript, through an intracellular-domain-independent mechanism. This study reveals a previously unknown interaction between two HSA21 genes that are important for axon development.

## RESULTS

### Increased *Appl* expression during development promotes axon terminal growth

**Fig. 1.**
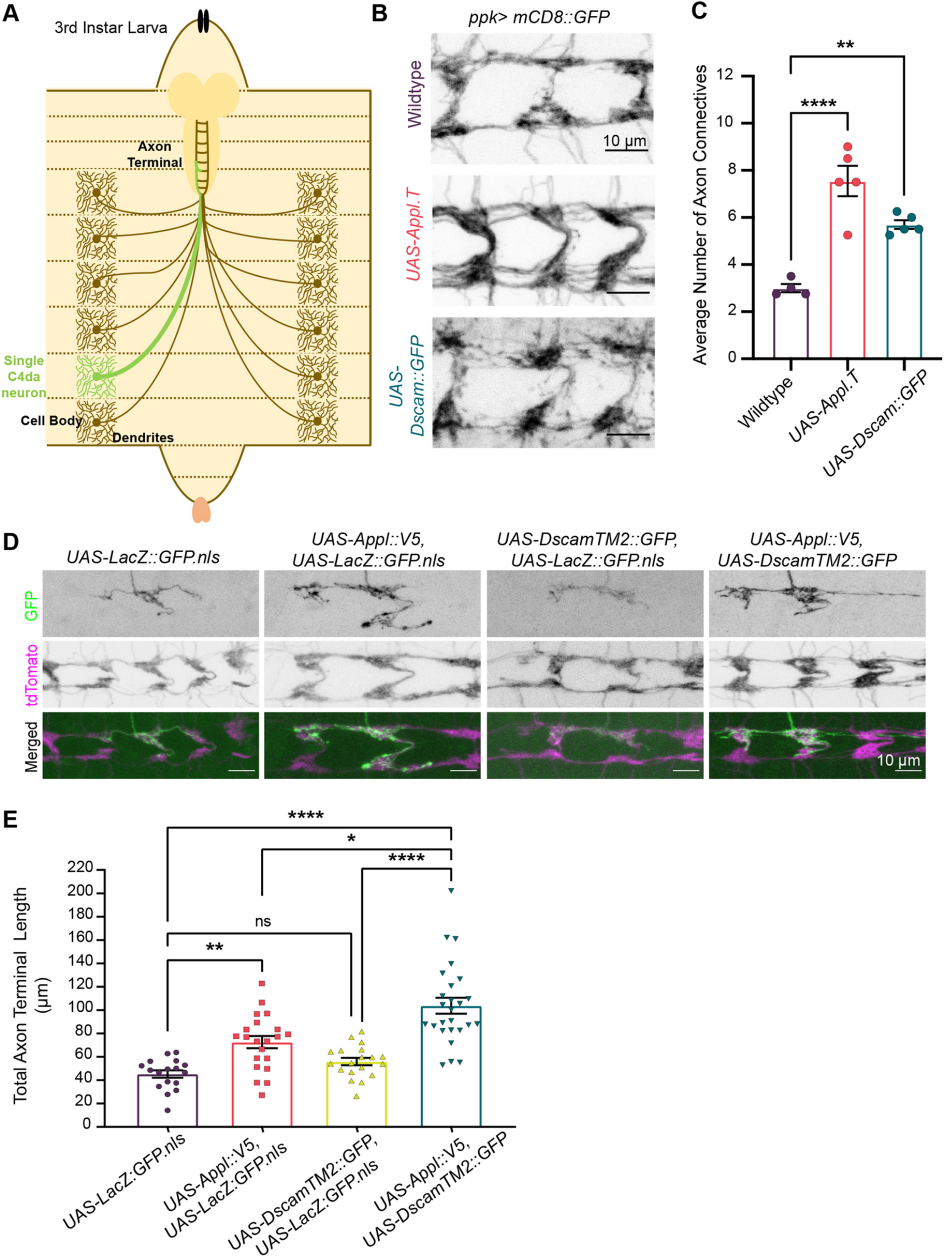
***Appl* loss and gain of function alters class IV dendritic arborization (C4da) axon terminal length.** (A) Schematic showing orientation of C4da neurons in *Drosophila* larvae. (B) Representative images of total axon terminals of C4da neurons. Overexpression of Appl or Dscam increases the number of axon projections compared to that in the wild-type control. For consistency, axon terminals in segments A4-A6 are shown and quantified throughout the paper. (C) Quantification shows a significant increase in the average number of axon connectives with *Appl* or *Dscam* overexpression. One-way ANOVA with Dunnett's multiple comparisons post hoc analysis. (D) Images compare a single axon terminal with overexpression of (1) *UAS-Appl::V5* and *UAS-LacZ::GFP.nls* (*n*=21 cells), (2) *UAS-DscamTM2::GFP* and *UAS-LacZ::GFP.nls* (*n*=19 cells), and (3) *UAS-Appl::V5* and *UAS-DscamTM2::GFP* (*n*=26 cells) compared to the control overexpression of *UAS-LacZ::GFP.nls* (*n*=17 cells), all on an *FRT^G13^, UAS-mCD8::GFP* background. (E) Quantification of D shows a significant increase in the length of single C4da axon terminals with co-overexpression of *DscamTM2::GFP* and *Appl::V5* compared to that in all other groups. *UAS*-site competition in the *Dscam*-only overexpressing group results in sufficiently decreased *Dscam* to show no axon overgrowth phenotype. This is distinct from results in Fig. 2A,C, in which fewer competing *UAS* sites were introduced and thus there was greater *Dscam* expression and a resulting axon overgrowth phenotype. One-way ANOVA with Tukey multiple comparisons post hoc analysis. ns, not significant (*P*>0.05), *P<0.05, **P<0.01, ****P<0.0001.

Class IV dendritic arborization (C4da) neurons provide an excellent model for studying neuronal morphology in *Drosophila* larvae (Grueber et al., 2003; Jan and Jan, 2003). The cell bodies and dendrites of these nociceptive neurons are located on the larval body wall, whereas their axons project into the ventral nerve cord (VNC) of the central nervous system (CNS) (Fig. 1A). This allows clear visualization of axon morphology separate from dendritic structures. In addition, stereotyped C4da axon terminal morphology allows reliable assessment of morphological changes. In the VNC, the axon terminal of each C4da neuron consists of anterior, posterior and, in some cases, contralateral branches (Grueber et al., 2007). These axon terminals, which are presynaptic arbors enriched with presynaptic markers (Kim et al., 2013), collectively form a ladder-like structure in the VNC (Grueber et al., 2003) (Fig. 1A,B). The anterior and posterior projections of the C4da axon terminals on each side of the ladder form a loose fascicle that connects two adjacent neuropils (Fig. 1B); the longitudinal axon terminals in this fascicle (i.e. the ‘axon connectives’) can be quantified (Sterne et al., 2015; Wang et al., 2013). We used this model to examine the morphological impact of altering expression levels of the *Drosophila* homologs *Appl* and *Dscam*.

**Fig. 2.**
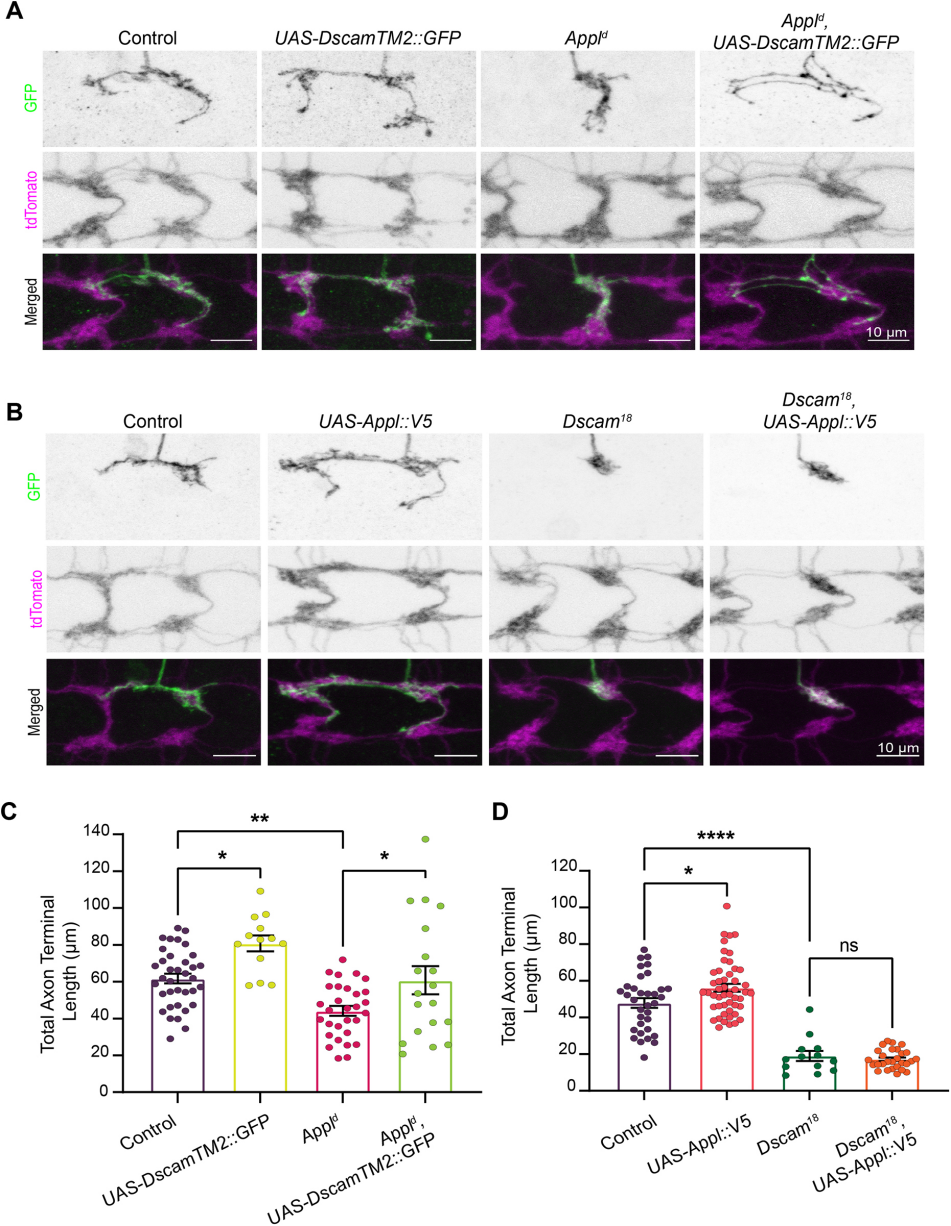
***Appl* requires *Dscam* to drive single C4da axon terminal growth.** (A,B) Representative images of the axon terminals of single C4da MARCM clones (GFP) contextualized in total C4da neuropil (tdTomato). All overexpression is driven with *ppk-Gal4*, and all backgrounds contain *UAS-mCD8::GFP* for clone selection. (A) Images compare a single axon terminal of (1) *UAS-DscamTM2::GFP* (*n*=13), (2) *Appl^d^* (*n*=30), and (3) *Appl^d^* and *UAS-DscamTM2::GFP* (*n*=19) to the control (*n*=36), all on an *FRT^19A^* background. (B) Images compare a single axon terminal of (1) *UAS-Appl::V5* (*n*=49), (2) *Dscam^18^* (*n*=13), and (3) *Dscam^18^* and *UAS-Appl::V5* (*n*=28) to the control *UAS-mCD8::GFP* (*n*=34), all on an *FRT^G13^* background. (C) Quantification of A shows a significant increase in the length of single C4da axon terminals with overexpression of *DscamTM2::GFP* in the absence of functional *Appl*. One-way ANOVA with Tukey multiple comparisons post hoc analysis. (D) Quantification of B shows no significant increase in the length of single C4da axon terminals with overexpression of *Appl::V5* in the absence of functional *Dscam*. One-way ANOVA with Tukey multiple comparisons post hoc analysis. ns, not significant (*P*>0.05), *P<0.05, **P<0.01, ****P<0.0001.

Developmental upregulation of APP in DS is well established (Cataldo et al., 2008), and prior work has shown that *Appl* gain of function leads to exuberant axon growth and synaptogenesis in adult *Drosophila* (Leyssen et al., 2005; Soldano et al., 2013). To determine whether the same is true in the larval system, we asked how increasing *Appl* expression during development affects C4da axon morphology. We used the Gal4/*UAS* system (Brand and Perrimon, 1993) to achieve C4da-specific overexpression of *Appl* transgenes using the *pickpocket* (*ppk*) promoter (Grueber et al., 2003).

We first looked at the number of axon connectives between two neuropils formed by C4da neurons in neighboring body segments to assess the effect of overexpressing Appl (Sterne et al., 2015; Wang et al., 2013). Overexpression of Appl produced significantly more axon connectives between two segments than were seen in wild-type flies, with an average of 7.6±0.64 (s.e.m.) axon connectives between two segments compared to 3.0±0.18 in wild type (Fig. 1B,C). This resembles the phenotype observed when overexpressing Dscam in these neurons (Kim et al., 2013). Dscam overexpression significantly increased the average number of axon projections per neuropil segment from 3.0±0.18 in wild type to 5.7±0.18 (Fig. 1B,C). This suggests that elevating the level of either Appl or Dscam leads to axon terminal overgrowth.

Because overexpression of *Dscam* and *Appl* individually resulted in comparable phenotypes, we next tested whether these two genes interact to regulate axon terminal length. To do this, we used mosaic analysis with a repressible cell marker (MARCM) to visualize single C4da neurons homozygous for mutations or overexpressing transgenes in an otherwise heterozygous or wild-type background (Lee and Luo, 1999). In addition to the transgenes of interest, a fluorescent cell membrane marker (*UAS-mCD8::GFP*) was expressed to label the morphology of single C4da neurons. Axon terminal length was measured from the axon branch point in the CNS to the axon endings (Kim et al., 2013). Using this technique, we compared single axon terminals in the following conditions: (1) overexpressing *UAS-mCD8::GFP* alone (negative control), (2) overexpressing only a V5-tagged *Appl* transgene (*UAS-Appl::V5*), (3) overexpressing only a GFP-tagged *Dscam* transgene (*UAS-Dscam::GFP*), and (4) overexpressing both *Appl::V5* and *Dscam::GFP*. In order to control for *UAS*-site competition for binding the GAL4 transcription factor, the negative control group, *Appl::V5-*only group and *Dscam::GFP-*only group also expressed an unrelated transgene – a LacZ::GFP fusion protein with a nuclear localization signal (*UAS-LacZ::GFP.nls*). This additional *UAS*-transgene in the *Dscam::GFP*-only group titrated *Dscam* expression to a level that did not drive significant changes in axon terminal length. Expressing *Dscam::GFP* at a non-phenotypic level allowed us to test whether Dscam co-expression with *Appl::V5* could drive more axon growth than expression of either construct independently.

Co-overexpression of *Appl* and *Dscam* increased axon terminal length more than expressing either transgene alone (Fig. 1D,E). This suggests that *Appl* and *Dscam* act additively to promote axon terminal growth. As it does not show whether these two genes function in a linear pathway or in parallel, we performed genetic epistasis tests to analyze the interaction between *Appl* and *Dscam*.

### *Appl* requires *Dscam* to promote C4da axon terminal growth

To determine whether *Appl* and Dscam function in a linear pathway or in parallel, we performed genetic epistasis tests to analyze the interaction between these two DS-related genes. To determine whether *Dscam* requires *Appl* to instruct axon terminal growth, we tested whether *Dscam* overexpression could increase axon terminal length in the absence of *Appl*. We used MARCM to examine the axon terminals of single C4da neurons in the *Appl* deletion mutant, *Appl^d^*, background (Torroja et al., 1999a). Loss of *Appl* significantly decreased axon terminal length by 28% (Fig. 2A,C), suggesting that *Appl* is required for establishing axon terminal length in C4da neurons during development.

As shown previously (Kim et al., 2013), *Dscam* overexpression in C4da neurons significantly increased axon terminal length (Fig. 2A,C). Importantly, overexpression of *Dscam* in *Appl^d^* neurons also increased axon terminal length compared to that in *Appl^d^* neurons without *Dscam* overexpression. *Dscam* overexpression in wild-type or *Appl^d^* neurons produced a comparable increase in axon terminal length (31% and 37%, respectively). Thus, *Dscam* can drive axon terminal growth without *Appl*, suggesting that *Appl* is not downstream of *Dscam*.

We also tested the possibility that *Dscam* is downstream of *Appl* by determining whether Appl overexpression increases axon terminal length in the absence of *Dscam*. As previously demonstrated by Kim et al. (2013), loss of *Dscam* (*Dscam^18^*) caused a decrease in axon terminal length (Fig. 2B,D). Moreover, *Appl* overexpression caused an expected increase in axon terminal length in the wild-type background (Fig. 2B,D). By contrast, *Appl* overexpression did not produce an increase in axon terminal length in the absence of *Dscam* (Fig. 2B,D). Therefore, *Appl* requires *Dscam* to drive axon terminal growth in C4da neurons. This indicates that *Dscam* functions downstream of *Appl*.

### *Appl* post-transcriptionally promotes Dscam protein expression

**Fig. 3.**
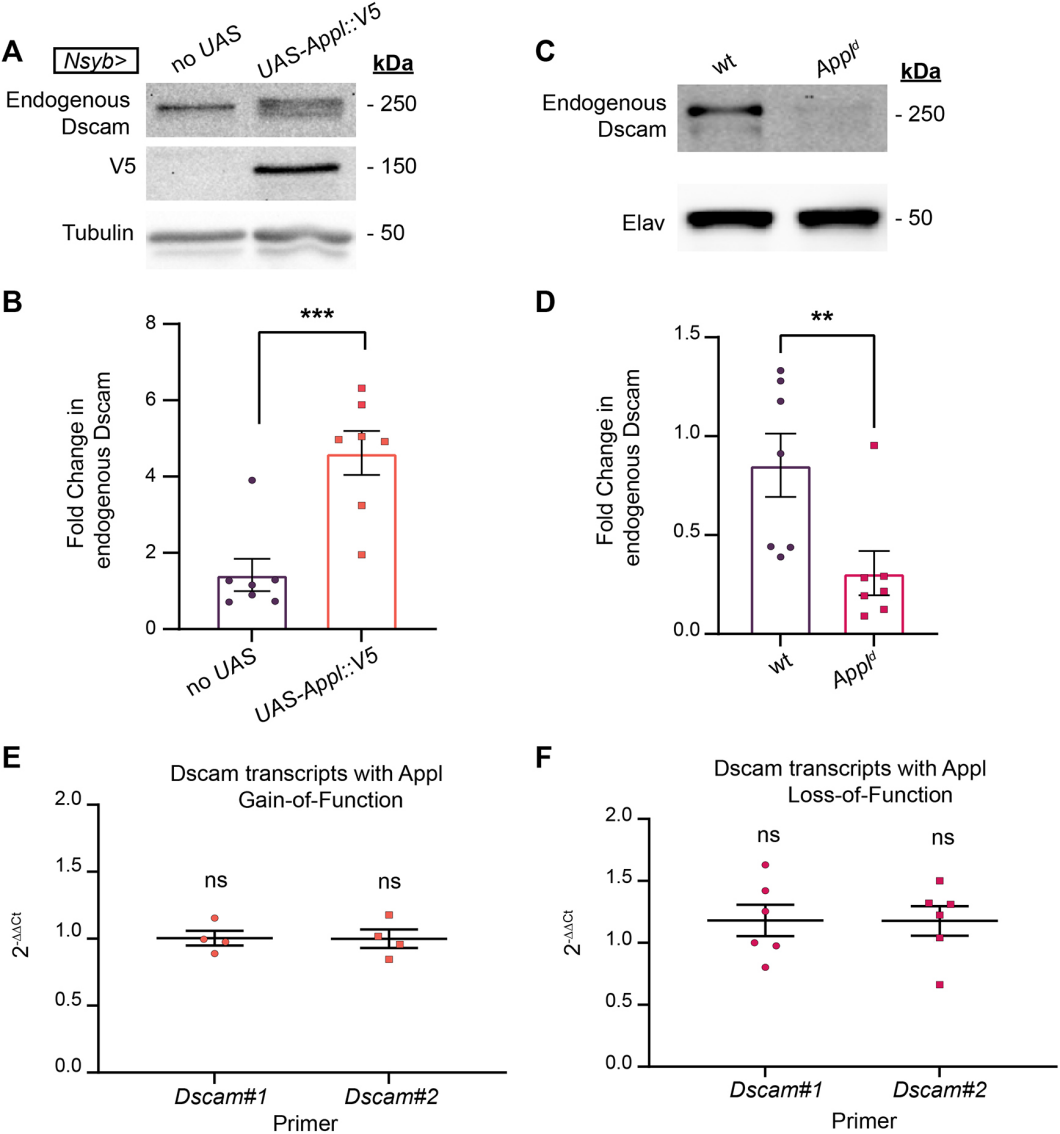
***Appl* modulates Dscam protein, but not mRNA, expression.** (A) Western blot staining for endogenous Dscam (250 kDa), Appl::V5 (150 kDa) and α-Tubulin (50 kDa, as an internal control for normalization) in the central nervous system (CNS) of larvae overexpressing Appl::V5 (*UAS-Appl::V5*) in all neurons (with *nSyb>Gal4*) or not (no UAS). (B) Quantification of A shows significant increase in endogenous Dscam expression with pan-neuronal (*nSyb>Gal4*) overexpression of *UAS-Appl::V5* compared to a ‘no *UAS*’ control (*w;;*). Two-tailed Mann–Whitney *U-*test, *n*=7 groups of 20 larval CNS per genotype, *P*=0.0023. (C) Western blot staining for endogenous Dscam (250 kDa) and Elav (50 kDa, as an internal control for normalization) in larval CNS that was either wild type or with loss of *Appl*. (D) Quantification of C shows a significant decrease in endogenous *Dscam* expression with loss of *Appl* (*Appl^d^*) compared to a wild-type control (*w;;*). Two-tailed Mann–Whitney *U-*test, *n*=6 groups of 20 larval VNCs per genotype, *P*=0.0070. (E,F) Quantification of fold change in endogenous *Dscam* mRNAs from larval CNS. The data were from RT-qPCR. No change was detected in endogenous *Dscam* transcript expression with gain (E) or loss (F) of *Appl*. Transcript expression was normalized internally to *Chmp1* (a reference gene) (Kim et al., 2013). Two separate primers (*DscamQ3* and *DscamQ5*) were used to catch all known *Dscam* isoforms. For each genetic manipulation and primer, significance was assessed. (E) 2^−ΔΔCt^ of *Dscam* transcripts for pan-neuronal overexpression of *Appl* (*nSyb>Appl::v5*) was then divided by the 2^−ΔΔCt^ control (*nSyb>mCD8::GFP*) to determine the fold change in transcripts. Two-tailed one-sample *t*-test, *n*=4 biological replicates per genotype, *P*=0.9493 (left) and *P*=0.9998 (right). (F) 2^−ΔΔCt^ of *Dscam* transcripts for total loss of *Appl* (*Appl^d^*) was then divided by the 2^−ΔΔCt^ control (*w;;*) to determine the fold change in transcripts. Two-tailed one-sample *t*-test, *n*=6 biological replicates per genotype, *P*=0.2132 (left) and *P*=0.1998 (right). ns, not significant (*P*>0.05), **P<0.01, ***P<0.001.

How might *Appl* require *Dscam* to promote axon terminal growth? Because Dscam expression levels linearly impact axon terminal length in C4da neurons (Kim et al., 2013), Appl might drive axon terminal growth by increasing Dscam expression. To determine whether *Appl* enhances Dscam expression, we tested the effects of *Appl* loss and gain of function on Dscam protein levels. We used early-third-instar larval CNS to examine endogenous Dscam protein levels because endogenous Dscam expression is higher and thus more easily detectable at this developmental timepoint. We used a pan-neuronal driver that expresses GAL4 under the *neuronal Synaptobrevin* (*nSyb*) promoter (*nSyb-GAL4*) to overexpress a V5-tagged *Appl* (*Appl::V5*), and then assessed proteins extracted from larval CNS via western blotting. Chemiluminescence of the protein bands was normalized to that of either α-Tubulin or Elav (a neuronal protein) (Kim et al., 2013). *Appl::V5* overexpression increased the level of endogenous Dscam compared to that of the no*-UAS* control (Fig. 3A,B). Conversely, loss of *Appl* (*Appl^d^*) decreased the amount of endogenous Dscam to one-third the amount observed in the wild-type control (Fig. 3C,D). By contrast, overexpression of Appl did not change the expression levels of Fasciclin 2 (Fas2; an immunoglobulin cell adhesion molecule in neurons) (Grenningloh et al., 1991), Fragile X messenger ribonucleoprotein 1 (Fmr1; an RNA-binding protein in neurons) (Zhang et al., 2001) or Cysteine string protein (Csp; a synaptic protein) (Zinsmaier et al., 1994) (Fig. S1). These results show that Appl positively regulates Dscam protein expression.

*Appl* may regulate Dscam protein expression transcriptionally or post-transcriptionally. To test whether *Appl* regulates Dscam expression at the transcript level, we extracted mRNA from the CNS of the early-third-instar larvae and determined transcript levels through real-time quantitative polymerase chain reaction (RT-qPCR). Two separate primer pairs (*Dscam*#1 and *Dscam*#2) that catch all known isoforms (Kim et al., 2013) were used to detect *Dscam* transcripts. We detected no change in *Dscam* transcript levels with pan-neuronal *Appl* overexpression (Fig. 3E) or *Appl* loss of function (Fig. 3F). This suggests that *Appl* regulates Dscam expression post-transcriptionally.

### *Appl* and its human homolog require extracellular domain 1 to promote axon terminal growth

**Fig. 4.**
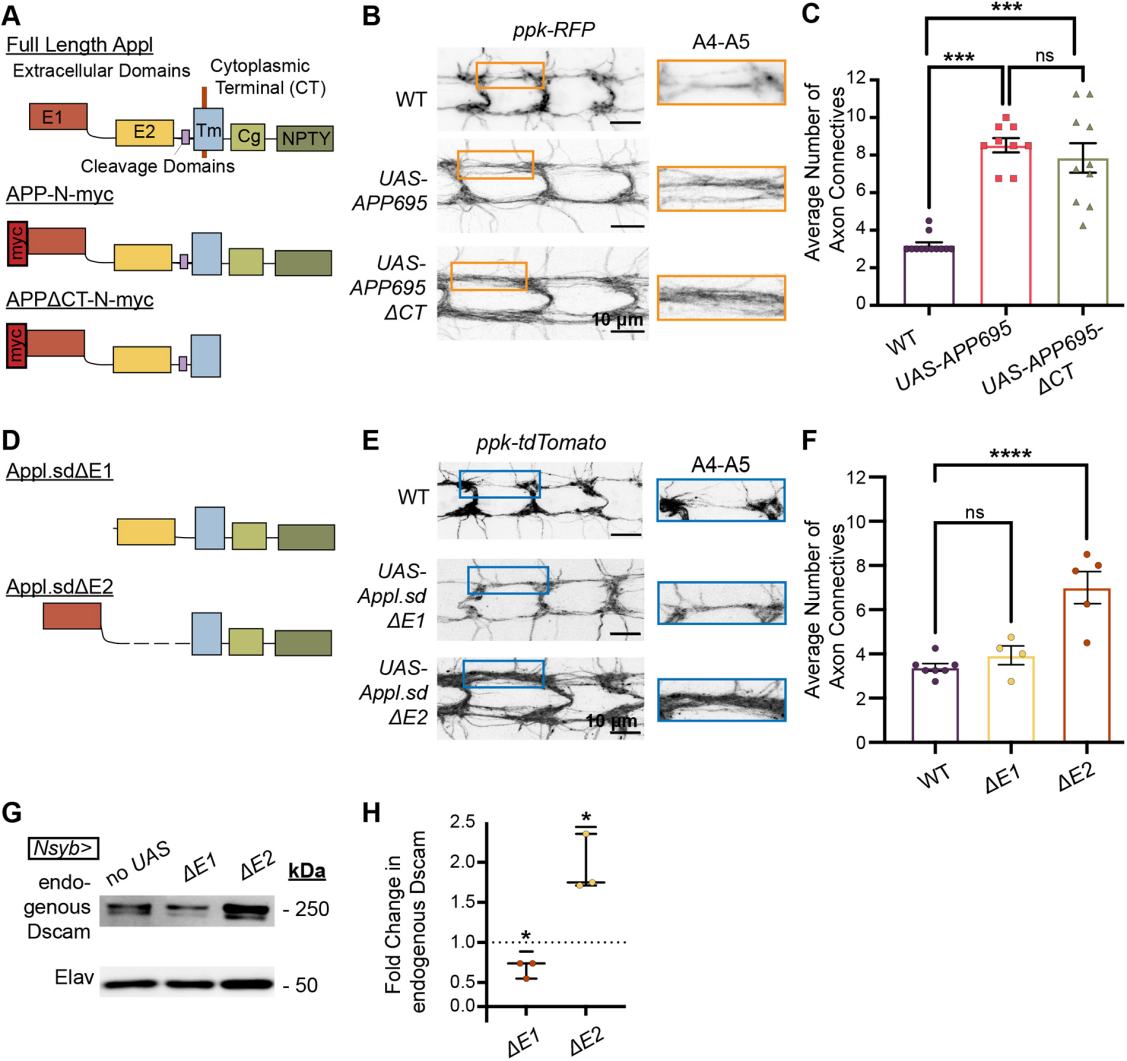
**Appl generally affects transmembrane protein expression through a secretion- and intracellular-domain-independent mechanism.** (A) Schematic of the domains in full-length Appl and the APP/Appl mutants used in B and C. E1, extracellular domain 1; E2, extracellular domain 2. (B) Axon terminals of all C4da neurons labeled by RFP fluorescence with (1) *ppk*>no overexpression (wild type; *w;;*), (2) *ppk*>UAS-APP695::myc and (3) *ppk*>UAS-APP695ΔCT::myc. The orange boxes show the magnified segments to the right for viewing the axon connectives, which were individually counted. (C) Average number of axon tracts describes the average of the total number of visible axon connectives between segments A4-A6. Quantification of B shows a significant increase in the number of axon connectives with C4da-specific overexpression of APP695 (UAS-APP695::myc) and APP695 lacking the cytoplasmic domain (UAS-APP695DCT::myc) compared to the control. Kruskal–Wallis with Dunn's Multiple comparisons post hoc analysis, *n*=9-10 per genotype. (D) Schematic of the domains in the Appl mutants used in E and F. The colors indicate the domains as labeled in panel A. (E) Total C4da neuropil labeled by tdTomato fluorescence with (1) *ppk*>no overexpression (wild type; *w;;*), (2) *ppk*>UAS-ApplΔE2 and (3) *ppk*>UAS-ApplΔE1. The blue boxes show the magnified segments to the right for viewing the axon connectives, which were individually counted. (F) Quantification of E shows a significant increase in the number of axon connectives when ApplΔE2, but not ApplΔE1, is expressed in C4da neurons. One-way ANOVA with Dunnett's multiple comparisons post hoc analysis. (G) Western blots of endogenous Dscam (250 kDa) and Elav (50 kDa) in the CNS of larvae overexpressing no UAS-*Appl* transgene (no UAS), UAS-Appl.sdΔE2 or UAS-Appl.sdΔE1 in all neurons (with *nSyb>Gal4*). (H) Quantification shows that Appl overexpression increases endogenous Dscam expression in the absence of the E2 domain and secretase binding sites, but not in the absence of the E1 domain. Two-tailed one-sample *t*-test, *P*=0.0096 (left) and *P*=0.0186 (right). ns, not significant (*P*>0.05), *P<0.05, ***P<0.001, ****P<0.0001.

To determine whether human APP and Appl share similar functions in regulating axon terminal growth, we studied the effect of human APP on C4da axon terminals. APP695 is the most common isoform in human neurons (Kang and Müller-Hill, 1990; Rohan de Silva et al., 1997). Prior work generated a *UAS* line driving Myc-tagged human *APP695* (Fig. 4A) (Mhatre et al., 2014). In APP695-overexpressing C4da, the average number of axon connectives between two segments of C4da neuropils was 2.7 times as many as that in the wild-type control (Fig. 4B,C). Thus, both *Drosophila* Appl and its human homolog APP696 produce axon overgrowth when overexpressed in C4da neurons.

We next sought to understand which domains of Appl/APP were required to drive axon growth. The intracellular domain of APP is important for regulating endosomal activity (Xu et al., 2016), transcription (Cao and Südhof, 2001; Scheinfeld et al., 2002; Zhao and Lee, 2003), cytoskeletal dynamics (Gertler et al., 1996; Müller et al., 2007; Sabo et al., 2003) and apoptosis (Haas et al., 1995; Lai et al., 1995; Weidemann et al., 1999). To determine whether the intracellular domain is required for APP to promote axon terminal growth, we expressed a transgene that encoded a human APP695 lacking the cytoplasmic domain (*UAS-APP695****Δ****CT*) (Fig. 4A) (Fossgreen et al., 1998) and compared its effect on C4da axon terminal growth with that of APP695 overexpression. Both transgenes caused robust overgrowth of axon terminals (Fig. 4B,C). This shows that the cytoplasmic domain is not required for APP to promote axon terminal growth.

Because the cytoplasmic domain of APP is not required to promote axon growth, we directly tested whether the *Appl*-driven axon terminal growth requires the *Appl* extracellular domains. APP and Appl have two highly conserved extracellular domains – extracellular domain 1 (E1) and extracellular domain 2 (E2) (Rosen et al., 1989). To determine whether one or both of these extracellular domains is required for *Appl* to instruct axon terminal growth, we expressed *Appl* mutant transgenes that lacked either E1 or E2 and then measured axon overgrowth. Both transgenes were secretion deficient (sd) and lacked the required binding sites for all known cleavage (Fig. 4D) (Torroja et al., 1999b). These transgenes are deletion mutants that produce peptides localized to the cell membrane but not the associated cleavage products released into the extracellular matrix (Fig. 4D) (Luo et al., 1992). Overexpression of the Appl construct lacking E2 (*UAS*-*Appl****Δ****E2*) still caused twice the number of average C4da axon tracts, compared to that in the control (Fig. 4E,F). By contrast, overexpression of the *Appl* construct lacking E1 (*UAS*-*Appl****Δ****E1*) did not cause detectable overgrowth. This suggests that Appl requires the E1, but not the E2, domain to drive C4da axon terminal growth.

Given that Appl overexpression increases Dscam levels post-transcriptionally and requires the E1 domain to promote axon terminal growth, we hypothesized that the Appl E1 domain is required to regulate Dscam expression. Thus, we tested whether selective loss of either the E1 or E2 domain of Appl interferes with Appl enhancement of Dscam levels. Consistent with the increased axon growth that occurs when *Appl.sdΔE2* is overexpressed, pan-neuronal overexpression of *Appl.sd****Δ****E2* increased endogenous Dscam protein levels (Fig. 4G,H). By contrast, overexpression of *Appl****Δ****E1* caused a mild, but consistent, decrease in endogenous Dscam expression (Fig. 4G,H), possibly due to disruption of endogenous Appl function. These results suggest that the Appl E1 domain is required for Appl overexpression to drive C4da axon overgrowth and for *Appl*-induced increases in Dscam expression.

### Appl overexpression does not affect Dscam protein degradation in cultured *Drosophila* cells

Because Appl alters Dscam protein expression without affecting *Dscam* transcript expression, we tested the possibility that Appl stabilizes Dscam protein. Results from co-immunoprecipitation (IP) experiments suggest that Dscam and Appl do not form a protein complex, as Appl was not pulled down together with Dscam (Fig. S2). Next, we tested whether Appl decreased Dscam protein turnover by examining Dscam protein levels in *Drosophila* Schneider 2 (S2) cells in the presence of the mRNA translation inhibitor cycloheximide (CHX). Dscam::GFP was co-expressed with either Appl or an exogenous membrane protein (CD8::GFP) as a negative control. CHX was then added to the cells to prevent further protein synthesis (Fig. S3). By sampling the cells in 4-h increments, we tested whether Dscam::GFP took longer to degrade in the presence of Appl overexpression. We did not observe a significant difference in the rate of Dscam::GFP degradation in the absence and presence of Appl overexpression (Fig. S3B,C). This result suggests that Dscam protein turnover is unlikely to explain the increase in Dscam expression by Appl.

### The effects of Appl and Dscam overexpression on nocifensive behavioral response

**Fig. 5.**
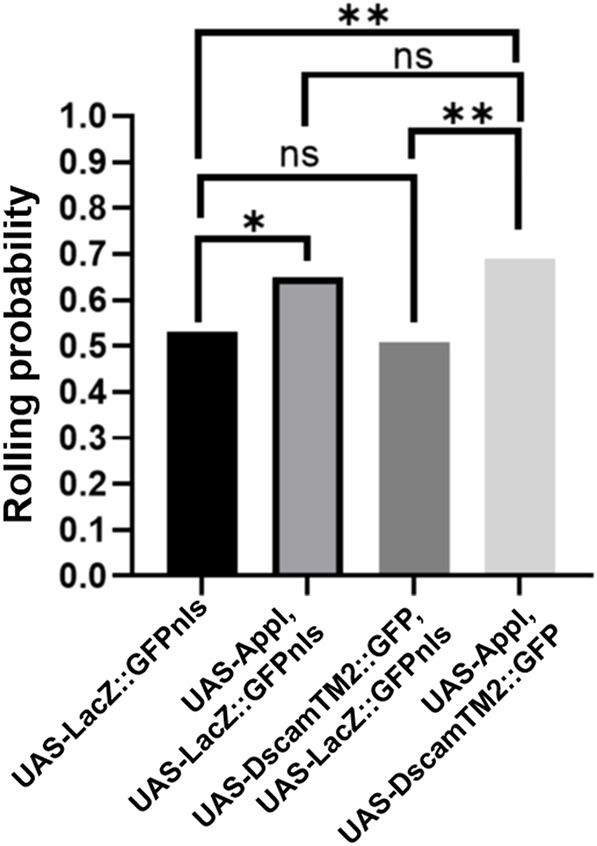
**The effects of Appl and Dscam overexpression on nocifensive behavioral response.** The graph shows the probability of heat-elicited nocifensive rolling in four genotypes of larvae: (1) *UAS-LacZ::GFP.nls* (*n*=145 larvae), (2) *UAS-Appl* and *UAS-LacZ::GFP.nls* (*n*=163 larvae), (3) *UAS-DscamTM2::GFP* and *UAS-LacZ::GFP.nls* (*n*=118 larvae), and (4) *UAS-Appl* and *UAS-DscamTM2::GFP* (*n*=145 larvae). Chi-square tests. ns, not significant (*P*>0.05), *P<0.05, **P<0.01.

We performed behavioral experiments to evaluate the effects of Appl and Dscam overexpression on the functions of C4da nociceptors. *Drosophila* larvae respond to noxious stimuli activating C4da nociceptors by rolling their bodies around the rostrocaudal axis (Hwang et al., 2007; Tracey et al., 2003). The C4da nociceptors are activated by heat (Hu et al., 2020; Tracey et al., 2003), harsh pokes (Guo et al., 2014; Kim et al., 2012) and noxious chemicals (Kaneko et al., 2017; Zhong et al., 2012). We stimulated the larvae with intermediate heat at 33°C (Hu et al., 2020). Nocifensive rolling response was recorded in negative control (*UAS-LacZ::GFP.nls*), Appl overexpression, Dscam overexpression and Appl–Dscam co-overexpression conditions. Similar to the experiments for evaluating axon terminal growth, the negative control group, Appl overexpression group and Dscam overexpression group also expressed UAS-LacZ::GFP.nls so that the number of UAS-transgenes in them was the same as that in the Appl-Dscam co-overexpression group. The additional UAS-LacZ::GFP.nls in the *Dscam* overexpression group titrated *Dscam* expression to a level that did not cause significant changes in axon terminal length (Fig. 1D,E). Consistently, expressing *Dscam* at this level did not cause significant change in the nocifensive rolling elicited by heat (Fig. 5). Overexpression of Appl led to a significant increase in nocifensive rolling, which is consistent with its effect in increasing axon terminal length (Fig. 1D,E). Co-overexpression of Appl and Dscam increased heat-elicited nocifensive rolling, an effect that was not significantly different from that of Appl overexpression alone (Fig. 5).

## DISCUSSION

Trisomy of HSA21 results in increased RNA and protein levels of many of the triplicated genes on HSA21 (Cheon et al., 2003; Olmos-Serrano et al., 2016). Although some genes have been found to be responsible for aspects of DS, interactions between HSA21 genes have just begun to be studied. For example, *DSCAM* cooperates with *COL6A2* in congenital heart defects (Grossman et al., 2011) and may interact with *BACE2* to increase susceptibility to Hirschsprung disease (Lu et al., 2021). In this study, we found that HSA21 homologs *Appl* and *Dscam* functionally interact during development to establish axon terminal length. *Appl* drives axon terminal growth by increasing the expression of Dscam at the protein, but not transcript, level. Furthermore, *Appl* requires E1, but not the intracellular domain or E2, to drive this axon phenotype.

APP promotes neurite outgrowth in a variety of settings (Hoe et al., 2009; Koo et al., 1993; Qiu et al., 1995; Small et al., 1994; Young-Pearse et al., 2008). APP expression is upregulated during the neuronal maturation of primary hippocampal cultures (Hung et al., 1992). This upregulation during development is also seen in *Drosophila*, in which Appl is enriched in areas of synapse formation and in growing axons (Torroja et al., 1996). Appl has been shown to play a role in axonal outgrowth in small lateral ventral neurons and mushroom body neurons in adult *Drosophila* (Leyssen et al., 2005; Soldano et al., 2013). These studies also suggest that, like Dscam (Sterne et al., 2015), Appl signals through Abl. We previously showed that Dscam binds to Abl via its cytoplasmic domain and activates this kinase (Sterne et al., 2015). Moreover, the axon terminal overgrowth caused by increased Dscam is ameliorated by genetic and pharmacological inhibition of Abl. The present study demonstrates that Appl positively regulates Dscam expression. This could explain why Appl requires Abl for axon growth (Leyssen et al., 2005).

Previous studies have shown that, in DS models, increased APP expression causes an increase in RAB5 activation (Xu et al., 2016), which creates enlarged early endosomes and may contribute to the AD commonly seen in DS patients (DS-AD) (Cataldo et al., 2003; Cattaneo and Calissano, 2012; Salehi et al., 2006; Xu et al., 2016). Cleavage products derived from the intracellular domain produces a peptide that enhances RAB5-related endosomal activity (Cataldo et al., 2003; Cattaneo and Calissano, 2012; Salehi et al., 2006; Xu et al., 2016). In this study, we found that Appl drives an increase in axon terminal growth independent of the intracellular domain. This suggests that either Appl does not modulate Rab5 activity to increase Dscam or it acts through a novel endosomal pathway.

We also show that the E1 domain of Appl is required to promote axon terminal growth. This domain has previously been shown to be important for activation of G_O_-protein (Okamoto et al., 1995) and for APP dimerization (Dahms et al., 2010). Further investigation of how E1 contributes to the regulation of Dscam and axon terminal growth could provide mechanistic insights into the APP–DSCAM interaction.

Appl overexpression in larval nociceptors increased the behavioral responses mediated by these neurons, indicating that the overgrowth of presynaptic terminals might enhance the synaptic transmission from nociceptors to the second-order neurons in the nociceptive pathway. However, co-overexpression of Appl and Dscam increased heat-elicited nocifensive rolling to a degree that was similar to that caused by overexpression of Appl alone (Fig. 5). There are many possible explanations for this. For example, it is possible that although Appl and Dscam are in a linear pathway for regulating axon terminal growth, their roles in synaptic transmission overlap but are not strictly linear. Future studies should examine the effects of Appl and Dscam overexpression on synaptic and circuit functions.

The present study could also provide insights into neurodegeneration in DS-AD. As might be expected with the well-known AD-related processing of APP into Aβ, 98% of DS patients develop Aβ plaques by the age of 40 (Coppus et al., 2006). Strong evidence supports the role of APP in DS-AD pathogenesis (Doran et al., 2017; Kleschevnikov et al., 2012, 2004; O'Doherty et al., 2005; Salehi et al., 2006; Wiseman et al., 2018). Prior works have shown that although APP triplication causes hippocampal Aβ plaque formation in mouse DS models, additional homologs of HSA21 genes increase the quantity of Aβ plaques (Wiseman et al., 2018), indicating that genetic interactions determine phenotypic severity in DS-AD. In the present study, we present Dscam as a functional interactor of Appl. Future studies should determine whether an APP–DSCAM interaction contributes to the pathogenesis of DS-AD.

## MATERIALS AND METHODS

### *Drosophila* genetics

*UAS-LacZ::GFP.nls* (Shiga et al., 1996), *w^118^* (Pastink et al., 1988), *Appl^d^* (Torroja et al., 1999a), *UAS-Appl.T* (Torroja et al., 1999a), *UAS-DscamTM2::GFP[2 3.36.25]* (Wang et al., 2004), *UAS-mCD8::GFP* (Lee and Luo, 1999), *Dscam^18^* (Wang et al., 2002)*, hsFLP^122^* (Campbell et al., 1993), *FRT^19A^* (Xu and Rubin, 1993), *FRT^G13^* (Lee and Luo, 1999), *ppk-RFP* (Han et al., 2011), *ppk-tdtomato* (Grueber et al., 2003), *ppk-Gal4* (Grueber et al., 2003), *yw;; nSyb-Gal4*, *UAS-APP695::myc* (Fossgreen et al., 1998), *UAS-APP695ΔCT::myc* (Fossgreen et al., 1998), *UAS-ApplΔE2* (Torroja et al., 1999b) and *UAS-ApplΔE1* (Torroja et al., 1999b) lines were used in this study.

### Generation of DNA constructs and fly lines

The *pUASTattB-Appl::V5* transgenic fly was generated by isolating the *Appl* sequence from *w^118^ Drosophila* and subcloning it into the pUASTattB-V5 vector using an InFusion cloning system (Clontech, Mountain View, CA, USA). The resulting pUASTattB-Appl::V5 transgene was then injected into embryos carrying the attP40 docking site for making transgenic lines. We generated constructs for S2 cell transfection by inserting the *Appl*, *CD8* and *Dscam* sequences into the pAc5.1-V5/His or the pAc5.1-GFP plasmid backbone.

### MARCM

Single presynaptic terminals were visualized with MARCM as previously described (Kim et al., 2013). *FRT^G13^* and *FRT^19A^* lines were heat shocked for 15 min, and no horseradish peroxidase (HRP) staining was performed. Axon terminals were measured using Neurolucida software (MBF Bioscience), and branches under 5 µm were excluded. *ppk-Gal4, hsFLP, UAS-mCD8::GFP; tub-Gal80, FRT^G13^* flies were mated with (1) *UAS-Appl::V5, FRT^G13^, UAS-LacZ::GFP.nls, FRT^G13^*; (2) *UAS-Appl::V5, UAS-LacZ::GFP.nls, FRT^G13^*; (3) *UAS-DscamTM2::GFP, UAS-LacZ::GFP.nls, FRT^G13^*; (4) *UAS-Appl::V5, UAS-DscamTM2::GFP, FRT^G13^*; (5) *Dscam^18^, FRT^G13^*; (6) *Dscam^18^*, *UAS-Appl::V5, FRT^G13^*; or (7) *FRT^G13^* flies (Fig. 1D,E and Fig. 2B,D). *ppk-Gal4, hsFLP, UAS-mCD8::GFP; tub-Gal80, FRT^19A^* flies were mated with: (1) *Appl^d^, FRT^19A^*; (2) *FRT^19A^*; (3) *UAS-DscamTM2::GFP, FRT^19A^*; (4) *Appl^d^, FRT^19A^*; or (5) *Appl^d^,UAS-DscamTM2::GFP, FRT^19A^* flies (Fig. 2A,C).

### Immunohistochemistry and confocal microscopy

Immunostaining of third-instar larvae was performed as previously described (Ye et al., 2011). Antibodies used included chicken anti-GFP (Aves Labs, Tigard, OR, USA), rabbit anti-RFP (Rockland Immunochemicals, Limerick, PA, USA) and mouse anti-Myc (Sigma Aldrich). After staining, larval body-wall fillets were dehydrated with a series of ethanol and xylene washes and then mounted with DPX mounting medium (Electron Microscopy Sciences, Hatfield, PA, USA). Confocal imaging was performed on a Leica SP5 confocal system equipped with a resonant scanner, 20× oil-immersion lens and 63× oil-immersion lens. Images were collected and quantified as previously described (Kim et al., 2013).

### Western blotting

Western blotting was performed on samples from the CNS (brain and VNC) of late-third-instar larvae or S2 cells as described previously (Kim et al., 2013). Briefly, samples were prepared in SDS sample buffer containing β-mercaptoethanol (one larval CNS per microliter of sample buffer) and run on 8% acrylamide gel on a Bio-Rad Mini-Protean Tetra Cell system. The proteins were transferred to nitrocellulose (Fig. 3) or PVDF (the blots in the other figures) membranes and blocked with 5% milk (wt/vol) in 1× Tris-buffered saline (TBS) containing Tween 20 (TBST) for 1 h at room temperature. The blots were incubated with primary antibodies overnight at 4°C. After three washes in 1× TBST for 10 min, the blots were incubated with HRP-conjugated secondary antibodies (1:5000) for 2 h. The blots were then washed three times for 10 min each in 1× TBST and rinsed briefly in 1× TBS. Blots were developed by chemiluminescence (Pierce, 32106) and imaged with a Bio-Rad Chemidoc. Pixel intensity was measured using Fiji/ImageJ software to determine the total arbitrary units under the curve for a given band. Chemiluminescence for all quantified protein bands was normalized to that of α-Tubulin or Elav.

Primary antibodies used were rabbit anti-GFP (gift from Dr Yang Hong; 1:3000) (Hong et al., 2003), mouse anti-V5 (Invitrogen, R960-25; 1:5000), mouse anti-α-Tubulin [Developmental Studies Hybridoma Bank (DSHB), 12G10; 1:5000], rat anti-Elav (DSHB, 7E8A10, 1:5000), mouse anti-Dscam (Exon18) (gift from Dr Tzumin Lee; 1:1000) (Shi et al., 2007), mouse anti-Fas2 (DSHB, 1D4; 1:100) (Grenningloh et al., 1991), mouse anti-Csp (DSHB, DCSP-2, 6D6; 1:100) and mouse anti-Fmr1 (DSHB, 5A11-C; 1:500). Secondary antibodies were HRP-conjugated anti-mouse, anti-rabbit and anti-rat (Jackson ImmunoResearch).

### S2 cell culture, transfection and protein degradation assay

S2 cells were purchased from *Drosophila* Genomics Resource Center (S2-DRSC). The vendor authenticated the cell line. S2 cells were cultured in Schneider's *Drosophila* medium (Gibco, 21720-024) supplemented with 10% heat-inactivated fetal bovine serum (FBS) at 28°C. Five-hundred microliters of cells were plated at a density of 0.8×10^6^ 24 h before transfection with home-made polyethylenimine (PEI). DNA plasmids were mixed with Opti-Mem solution (Thermo Fisher Scientific), and PEI was used at a ratio to DNA of 1:5. Forty-eight hours after transfection, S2 cells were collected in ice-cold 1× PBS, centrifuged at 200 ***g*** for 2.5 min to remove medium and suspended in 50 µl 2× SDS sample buffer containing β-mercaptoethanol. Cells were mechanically disrupted and sonicated prior to western blotting.

For the protein degradation assay, CHX was dissolved in a minimal amount of dimethyl sulfoxide and added to each well at time zero (T0) at a concentration of 0.5 ng/µl. Cells in each well were collected individually at 4-h time increments, as shown in Fig. S3A, and frozen immediately at −25°C to prevent degradation.

### Co-IP

Forty-eight hours after transfection, S2 cells were solubilized in the IP buffer [150 mM NaCl, 1 mM CaCl_2_, 50 mM Tris-HCl (pH 7.4), 1% Triton X-100, 0.5 mM phenylmethylsulfonyl fluoride (PMSF) and EDTA-free protease inhibitor cocktail (Research Products International)]. The supernatant (300 µl) was incubated with anti-GFP antibody-conjugated magnetic beads (MBL, D153-11; 50 µl bead suspension) that were prepared according to the manufacturer's instructions. After incubating the cell lysates with the magnetic beads for 3 h at 4°C, the beads were washed three times in the IP buffer (5 min each) and three times in 1× PBS containing PMSF. The proteins bound to the beads were then eluted in 2× sample buffer without β-mercaptoethanol. The eluates were transferred to a new tube, before β-mercaptoethanol was added, and boiled for 5 min before loading to SDS-PAGE gels and analyzed with western blotting.

### RT-qPCR

RT-qPCR was performed on early-third-instar larval CNS as described previously (Kim et al., 2013). *Chmp1* was used as the reference gene. Two sets of primers were used to catch all known *Dscam* isoforms. Analysis was performed using a QuantStudio 5 Real-Time PCR Systems with Design and Analysis software 2.5 (Thermo Fisher Scientific). The primer sequences were as follows (Kim et al., 2013): *Chmp1*, 5′-AAAGGCCAAGAAGGCGATTC-3′ and 5′-GGGCACTCATCCTGAGGTAGTT-3′; *Dscam*#1, 5′-CTTACGATTGTGCTCATTACTC-3′ and 5′-CAGTTTCGATTTGTTCTGTTGG-3′; *Dscam*#2, 5′-ATCGAAACTGTTCAATGCAC-3′ and 5′-CTTGAGTGTATCTGTGTTTCGG-3′.

### Behavioral tests

Nocifensive behavioral tests were performed on mid-third-instar larvae. Larvae of different genotypes were tested on the same experimental day. In each trial, eight to ten larvae were placed on a thermoelectrically controlled heat plate (TC-720, TE Technology). The plate was covered with 750 µl water and pre-heated to 33°C. Behavioral responses were video recorded for 60 s. Videos were analyzed by LabGym software (Hu et al., 2023).

### Statistical analysis

All statistical analysis was performed using GraphPad Prism. Researchers were unaware of genotype/group when performing analyses. Normality was assessed for all groups. For data with normal distribution, two-group comparisons were made using an unpaired two-tailed *t*-test, and multiple group comparisons were made using one-way ANOVA with Tukey multiple comparisons post hoc analysis. For data with non-normal distribution, two-group comparisons were made using two-tailed Mann–Whitney *U-*test, and multiple group comparisons were made using Kruskal–Wallis with Dunn's multiple comparisons post hoc analysis. For normal data compared to a theoretical value, such as ratios where the null hypothesis would be a ratio of 1, one-sample *t*-tests were applied. For behavioral data, two-group comparisons were made using two-sided Chi-square tests. *P*<0.05 was considered significant.

## Supplementary Material

10.1242/dmm.049725_sup1 Supplementary information
