# Supplementary material for: The Drosophila homolog of APP promotes Dscam expression to drive axon terminal growth, revealing interaction between Down syndrome genes
Source: Dis Model Mech. 2023 Sep 15;16(9):dmm049725. doi: 10.1242/dmm.049725 (PMC10508694; doi:10.1242/dmm.049725)
Supplement: Supplementary information [file dmm-16-049725-s1.pdf]

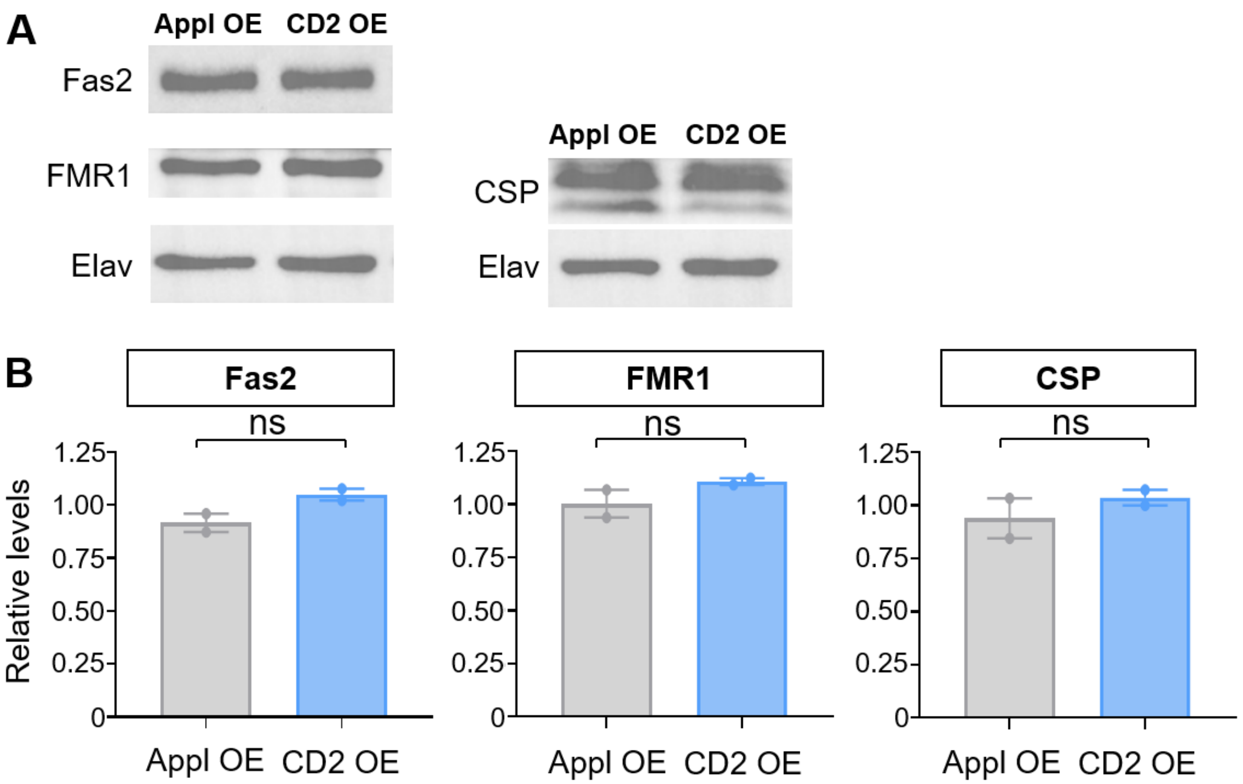

Fig. S1. ***Appl*** **modulates Dscam protein but not mRNA expression.**

UAS-Appl or UAS-CD2 (a type I transmembrane protein as a negative control) was expressed in all neurons by nsyb>GAL4. (**A**) Western blots for endogenous Fas2, FMR1, and CSP. The pan-neuronal protein Elav was used to normalize the samples. **(B)** Quantification and statistical analysis of the Western blots. Two-tailed Mann-Whitney U Test. ns: $p > 0.05$.

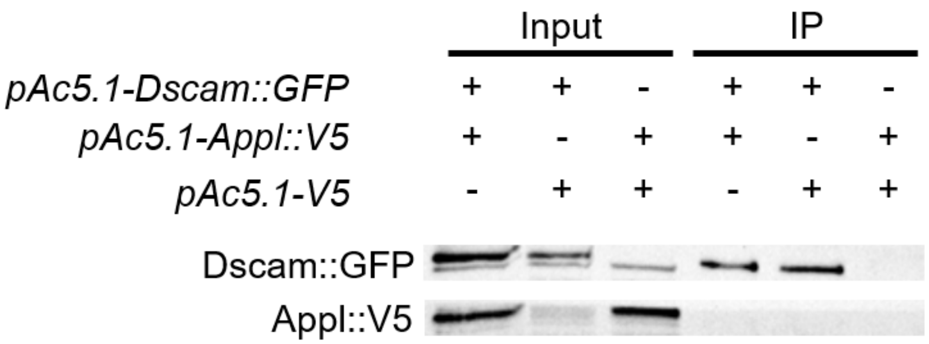

Fig. S2. Results from co-immunoprecipitation experiments suggest that Dscam and Appl do not form a protein complex.

S2 cells were transfected with *pAc5.1-Dscam::GFP* and *pAc5.1-Appl::V5* together, *pAc5.1-Dscam::GFP* alone, or *pAc5.1-Appl::V5* alone. Inputs show the expression of Dscam::GFP and Appl::V5 in the cell lysates. In the co-IP experiment, Dscam::GFP was immunoprecipitated (IP) with anti-GFP antibodies from the cell lysates, and then the immunoprecipitates were tested for the presence of Dscam::GFP and Appl::V5 by western blotting. Shown are representative western blots of one co-IP experiment. Whereas Dscam::GFP was successfully immunoprecipitated from cells expressing both Dscam::GFP and Appl::V5, as well as those expressing Dscam::GFP alone, Appl::V5 was not co-immunoprecipitated with Dscam::GFP. The same result was replicated in three different experiments.

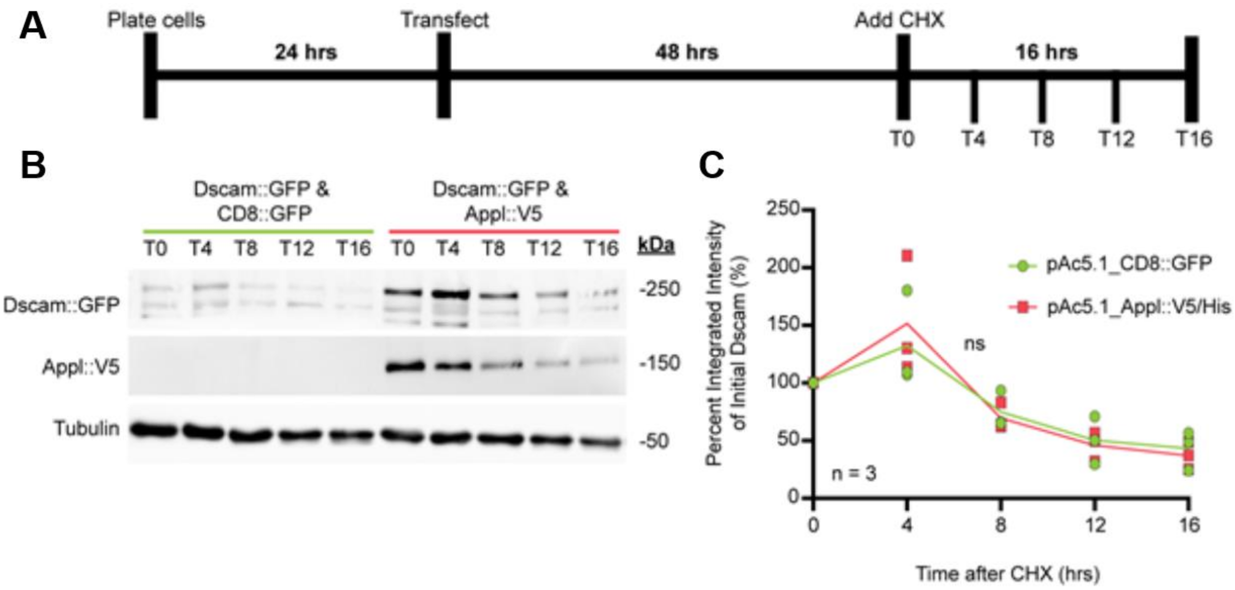

Fig. S3. Appl overexpression does not change the rate of Dscam protein degradation in S2 cells.

(A) Experimental timeline for the degradation assay to assess the rate of Dscam protein degradation in S2 Cells after the addition of the translation inhibitor cycloheximide (CHX). T0 is the timepoint at which CHX was added. T4, T8, T12, and T16 are 4, 8, 12, and 16 hours after CHX was added.

(B-C) Representative western blots (B) and quantification (C) showing the amount of Dscam::GFP detected from S2 Cell cultures collected at different timepoints after CHX treatment. The intensities of Dscam bands were normalized to those of tubulin bands at each timepoint. The percentage of integrated intensity was calculated by dividing the normalized Dscam intensity at a given timepoint by that at T0. No difference was detected in the presence and absence of Appl::V5 when comparing the amount of Dscam::GFP relative to the initial timepoint. Holm-Šídák Multiple t-tests. ns: $p > 0.05$.

Disease Models & Mechanisms • Supplementary information
